# Establishing new grasslands on crop fields: short‐term development of plant and arthropod communities

**DOI:** 10.1111/rec.13641

**Published:** 2022-03-03

**Authors:** Raja I. Hussain, Manuela Brandl, Bea Maas, Bernhard Krautzer, Thomas Frank, Dietmar Moser

**Affiliations:** ^1^ Institute of Zoology, Department of Integrative Biology and Biodiversity Research (DIB) University of Natural Resources and Life Sciences Vienna Austria; ^2^ Division of Conservation Biology, Vegetation and Landscape Ecology, Department of Botany and Biodiversity Research University of Vienna Vienna Austria; ^3^ Institute of Plant Production and Cultural Landscape Federal Research Institute Gumpenstein Irdning Austria

**Keywords:** arthropod diversity, new grassland strips, plant communities, restoration measures, seed mixtures, semi‐natural grassland

## Abstract

Establishment of semi‐natural grasslands offers a valuable approach to the conservation of threatened grassland biodiversity. We established new grassland strips on former crop fields adjacent to old semi‐natural grasslands and monitored the development of plant, carabid, spider, and wild bee communities over 3 years. The studied plant and arthropods communities were significantly different between newly established grassland strips and old grassland. Our results suggest that restoring plant and arthropod communities takes longer than 3 years to become similar to old semi‐natural grasslands.

**Conceptual Implications**

- Establishment of new grassland is a long‐term continuous process.
- Some arthropod groups (such as carabids) are faster in convergence than plants.
- The development of newly established grassland toward old grassland takes more than 3 years.

## Introduction

Semi‐natural grasslands are among the most diverse ecosystems in agroecosystems and offer a widespread range of ecosystem services (Feurdean et al. 2018). Yet, semi‐natural grasslands are increasingly endangered and fragmented due to land consolidation, intensification, and abandonment (White & Roy 2015). Moreover, deterioration of remaining semi‐natural grassland due to nitrogen input and changes in mowing frequency is reducing species richness of plants (Williams & Osborne 2009) and habitat availability for arthropods (Steffan‐Dewenter & Schiele 2008).

European countries launched specific agri‐environmental schemes to conserve semi‐natural grassland and counter the loss of arthropod diversity (Batáry et al. 2015). Yet, despite all efforts, habitat destruction and deterioration has progressed strongly during the last decades producing a heavily fragmented pattern of semi‐natural grassland remnants. Establishing new grassland, with native seed mixture, might be a tool (FAO 2021) to restore disrupted meta‐populations of semi‐natural grassland communities (Ouvrard et al. 2018). Such new grasslands are recognized to support arthropods (Hussain et al. 2021; Maas et al. 2021; Scharnhorst et al. 2021), but their efficiency strongly depends on plant communities (Schaffers et al. 2008). It is well known that plant communities determine the niche, food resource, and physical structure in grassland and, therefore, considerably influence the communities, species richness, and abundance of arthropods (McCoy & Bell 1991; Liu et al. 2015).

Many studies critically discussed the pattern and distribution of arthropods, but the overall development of plant communities in newly established grassland was mostly neglected (e.g. Korpela et al. 2013; Tschumi et al. 2015). However, the question of whether such newly established grasslands are restored to a high conservation value comparable to semi‐natural grassland has fundamental importance during any restoration attempt (Mitchell et al. 2000). The present work investigates the initial phase of newly established grasslands in crop fields dominated by winter wheat crops. We tested whether they are a promising supplement to already established old grassland and existing measures to maintain and protect agricultural biodiversity. We monitored plant and arthropod (spiders, carabids, and wild bees) communities in newly established grassland over 3 years, questioning: to what extent do species communities in newly established grassland differ from communities in old semi‐natural grassland in the course of 3 years?

## Methods

### Study Region

This study was conducted in an agricultural region of Lower Austria (Tullnerfeld 48°16′02.5″N 16°05′07.9″E, 48°15′08.3″N 16°02′56.9″E), located 55 km west side of Vienna, Austria. The study region is a mosaic of small patches of low‐intensity semi‐natural meadows, and conventionally managed crop fields. The region borders the forests of the Wienerwald Biosphere Reserve. The region has 9.9°C mean annual air temperature, 673 mm mean annual precipitation, and a mean elevation of 250 m.

### Establishment of New Grassland and Plant Sampling

**Figure 1:**
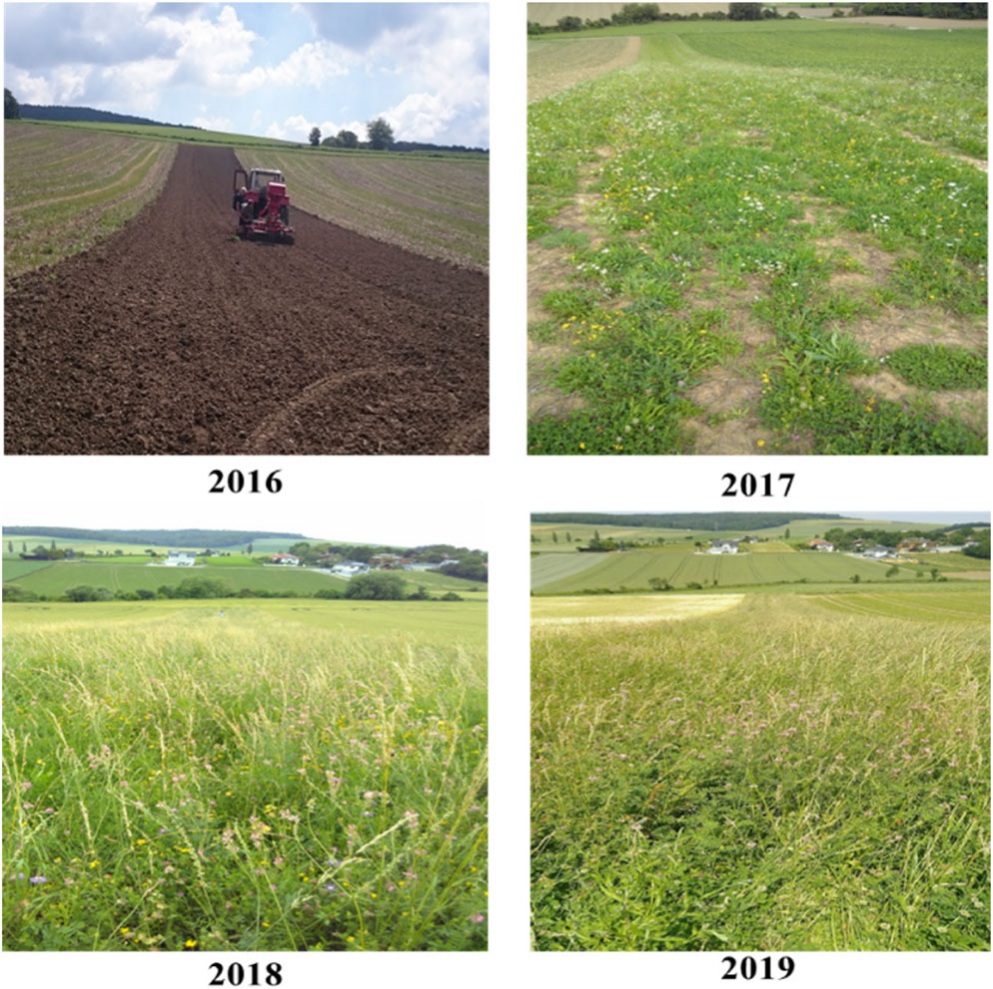
Establishment and development of newly established grasslands in 3 years. Different stages of grassland establishment are shown, that is from seed bed preparation till development.

Our experimental setup included five permanent semi‐natural grasslands (hereafter “old grassland” or “OG”) and five new grassland strips (250 × 10 m length; hereafter “NG”) established adjacent to the OG. The new grasslands were established on former crop fields. The distance between the newly established grasslands was 1,000 m on average. In August 2016, we established the NGs, using a seed mixture of 41 different plant species (with 14.65% legumes 51.35% herbaceous plants and 34.10% grass species) mimicking the plant composition of previously investigated meadows from the study region. For this, we analyzed 28 releves of Filipendulo‐Arrhenatheretum meadows and 54 releves of Ranunculo bulbosi‐Arrhenatheretum meadows (Hülber et al. 2017). Based on these samples, we selected the 50 most frequent plant species (i.e. occurring in more than 25% of the samples) and selected a final set of 41 final plants based on availability of seed material (Tables S1–S3). After establishment, the NG sites were managed by one mowing event per year in late summer. However, OG sites were mown twice per year (Fig. 1).

For monitoring plant development, we recorded plant species within 2 × 2–m observation plots in May (i.e. when most of the plants were blooming) for three consecutive years (2017–2019). Plant abundance was measured using the Braun‐Blanquet scale (Dengler et al. 2008) translated into percentage cover, and taxonomy following Fischer et al. (2008). Observation plots were placed within OG and in NG at 35, 70, 105, 140, and 175 m distance from OGs so the whole sample consisted of 6 observation plots × 5 study sites = 30 plots per year.

### Carabid, Spider, and Wild Bee Sampling

In each sampling transect of NG, carabids and spiders were collected using two pitfall traps per plot at 35, 70, 105, 140, and 175 m from the OG for three consecutive years (2017–2019). At the same distances, wild bees were surveyed by selecting five 2‐m^2^ plots per transect using an observation plot method (Hussain et al. 2018). Equal numbers of pollinator sampling plots were made in OG irrespective of distances. Carabids, spiders, and wild bees were identified to species level by specialized taxonomists. In total, three pitfall trap surveys were carried out at 2 weeks interval between April and May in each year. However, four observation plot surveys were carried out for wild bees between May and August in each year.

### Statistical Analysis

**Figure 2:**
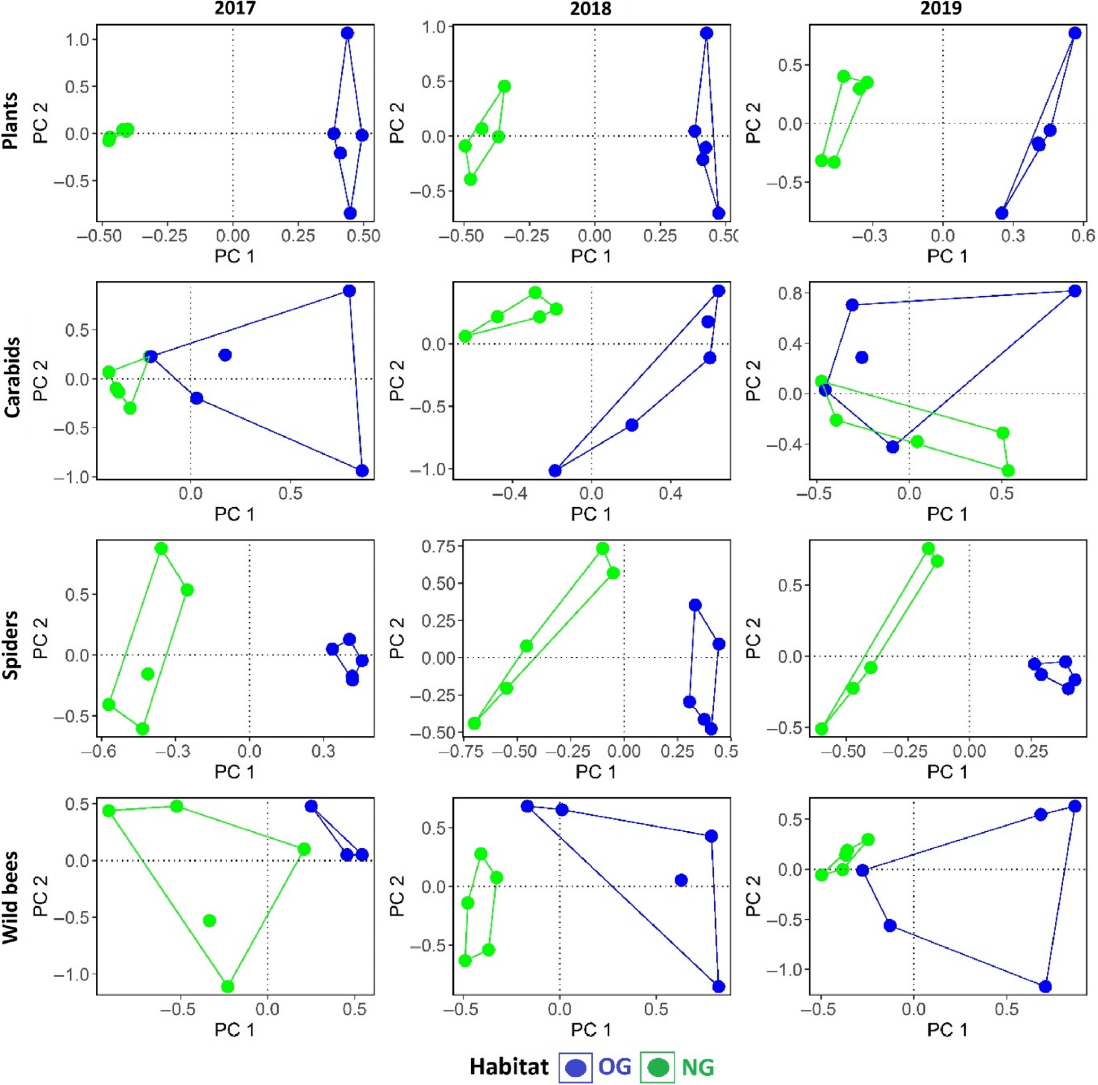
Ordination plot of principal component analysis showing the species communities between newly established grasslands (NG) and old grasslands (OG) in 3 years.

All statistical analyses were performed with the R program version 3.5.1 (R Core Team 2018). We used a multivariate approach to assess the development of plant and arthropod communities in NGs compared to OGs. For multivariate analysis, the data from each sampling year were pooled and standardized using the “Hellinger” transformation (Sławska et al. 2017). Permutational analysis of variance (PERMANOVA), from the function “adonis,” was used to assess the change in species communities between NG and OG by taking year as an explanatory variable in the model. The Bray–Curtis dissimilarity measure was used as a distance metric with 999 permutations for the probability tests. Data were tested for equal multivariate dispersion using the function “betadisper.” The outcome of the betadisper analysis was visualized using boxplots (Fig. S1). For visualization of the community change, we present ordination plot of principal component analysis in Figure 2.

## Results and Discussion

The plant communities in NG were significantly different over the observed 3‐year period. The total number of new plant species in NG increased from 41 sown species, at the stage of sowing, to 68 in 2017, 47 in 2018, and 53 in 2019. The increase in 2017 could be attributed predominantly to annuals that decreased in 2018 and were replaced by perennial invaders. A total of 221 arthropods species, including 58 species of carabids, 73 species of adult spiders, and 66 species of wild bees, were recorded. The total number of recorded individuals was 11,022, including 3,615 carabids, 7,072 spiders, and 365 wild bees. The average distance of the samples to the group centroid from the multivariate homogeneity test (betadisper) was significantly different between studied arthropods and plants. Further, it showed that both the arthropods and plants had a greater variation in NG and OG. Plant, carabid (except in 2019), spider, and wild bee species communities were significantly different between NG and OG in all 3 years.

Plants and arthropods are among the most diverse species groups, contributing pivotal ecological and evolutionary trophic interactions to grasslands and surrounding ecosystems (Futuyma & Agrawal 2009; Forister et al. 2012). In the first year, plant cover was sparse with seedlings unevenly distributed, high cover of weeds, and large patches of bare ground. However, after the first mowing, particularly grasses started to gain in abundance and increasingly dominated the vegetation. We established the grasslands on crop fields with particularly high nutrient supply, which was build up over years of intensive use. Consequently, competition between sown plants intensified and resulted in a change of species communities. Further, mowing frequency had a greater impact on the increase in plant species abundance (Sehrt et al. 2020). Such changes in plant communities determine the availability of both structural niches and food resources required by different carabids (Asteraki et al. 2004; Axmacher et al. 2009), spiders (Lafage et al. 2019), and wild bees (Cusser & Goodell 2013).

Our results demonstrate species communities in newly established grasslands only converge very slowly to old grassland communities, and 3 years are not nearly enough to reach convergence. This might be due to three main reasons: (1) Succession in arthropods (except carabid) lagged behind succession in plants since competition between plants changes habitat quality for arthropods (Seibold et al. 2019); (2) Establishment of arthropods communities also depended on natural colonization from the surrounding landscape (Collinge 2000); and (3) Generalist arthropods colonized rapidly, while specialist arthropods might have suffered competition from these earlier arrivals, which delays succession to several years (Snyder & Evans 2006).

Overall, the communities of studied taxonomic groups significantly differed between OG and NG in all 3 years (except carabids in 2019) and it might take several more years until plant and arthropod communities of such newly established grasslands really get similar to the old meadows.

## Supporting information


**Figure S1.** Box and whiskers plot showing the variation in the distribution of distance to centroid of pairwise comparisons among newly established grassland (NG) and old grassland (OG).
**Table S1.** List of plant species sown in newly established grasslands (NG).
**Table S2.** List of studied arthropods abundance and richness in old grassland (OG) and newly established grassland (NG).
**Table S3.** Results from the PERMANOVA (“adonis”) among studied habitats (newly established grassland and old grassland) based on Bray‐Curtis dissimilarity with 999 permutations.
