# Supplementary material for: Establishing new grasslands on crop fields: short‐term development of plant and arthropod communities
Source: Restor Ecol. 2022 Mar 3;30(8):e13641. doi: 10.1111/rec.13641 (PMC9790339; doi:10.1111/rec.13641)
Supplement: Supplementary file 1 — Figure S1. Box and whiskers plot showing the variation in the distribution of distance to centroid of pairwise comparisons among newly established grassland (NG) and old grassland (OG). Table S1. List of plant species sown in newly established grasslands (NG). Table S2. List of studied arthropods abundance and richness in old grassland (OG) and newly established grassland (NG). Table S3. Results from the PERMANOVA (“adonis”) among studied habitats (newly established grassland and old grassland) based on Bray‐Curtis dissimilarity with 999 permutations. [file REC-30-0-s001.docx]

**Supporting information**

Table S1. List of plant species sown in newly established grasslands (NG).

| **S. NO.** |  | **Family** | **Species name** |
| --- | --- | --- | --- |
| 1 | Grasses | Poaceae | *Anthoxanthum odoratum* |
| 2 |  | Poaceae | *Arrhenatherum elatius* |
| 3 |  | Poaceae | *Brachypodium pinnatum* |
| 4 |  | Poaceae | *Briza media* |
| 5 |  | Poaceae | *Bromus erectus* |
| 6 |  | Poaceae | *Festuca pratensis* |
| 7 |  | Poaceae | *Festuca rubra agg.* |
| 8 |  | Poaceae | *Festuca rupicola* |
| 9 |  | Poaceae | *Holcus lanatus* |
| 10 |  | Poaceae | *Koeleria pyramidata* |
| 11 |  | Poaceae | *Poa pratensis agg.* |
| 12 | Legumes | Fabaceae | *Anthyllis vulneraria* |
| 13 |  | Fabaceae | *Lotus corniculatus* |
| 14 |  | Fabaceae | *Medicago lupulina* |
| 15 |  | Fabaceae | *Onobrychis viciifolia* |
| 16 |  | Fabaceae | *Trifolium pratense* |
| 17 |  | Fabaceae | *Trifolium repens* |
| 18 | Herbs | Asteraceae | *Achillea millefolium* |
| 19 |  | Asteraceae | *Buphthalmum salicifolium* |
| 20 |  | Campanulaceae | *Campanula patula* |
| 21 |  | Asteraceae | *Centaurea jacea* |
| 22 |  | Asteraceae | *Centaurea scabiosa* |
| 23 |  | Asteraceae | *Centaurea stoebe* |
| 24 |  | Asteraceae | *Crepis biennis* |
| 25 |  | Asteraceae | *Daucus carota* |
| 26 |  | Caryophyllaceae | *Dianthus carthusianorum* |
| 27 |  | Rubiaceae | *Galium mollugo* |
| 28 |  | Rubiaceae | *Galium verum* |
| 29 |  | Hypericaceae | *Hypericum perforatum* |
| 30 |  | Caprifoliaceae | *Knautia arvensis* |
| 31 |  | Asteraceae | *Leontodon hispidus* |
| 32 |  | Asteraceae | *Leucanthemum vulgare* |
| 33 |  | Plantaginaceae | *Plantago lanceolata* |
| 34 |  | Plantaginaceae | *Plantago media* |
| 35 |  | Lamiaceae | *Prunella grandiflora* |
| 36 |  | Ranunculaceae | *Ranunculus bulbosus* |
| 37 |  | Polygonaceae | *Rumex acetosa* |
| 38 |  | Lamiaceae | *Salvia pratensis* |
| 39 |  | Rosaceae | *Sanguisorba minor* |
| 40 |  | Caryophyllaceae | *Silene nutans* |
| 41 |  | Caryophyllaceae | *Silene vulgaris* |

Table S2. List of studied arthropods abundance and richness in old grassland (OG) and newly established grassland (NG). Abundance is given in numbers, while empty space indicates absence.

| **S. NO** | **Species name** | **Old grassland(OG)** | **Newly established grassland (NG)** |
| --- | --- | --- | --- |
| **Carabids** | |  |  |
| 1 | *Abax carinatus* | 1 |  |
| 2 | *Abax parallelepipedus* |  | 1 |
| 3 | *Acupalpus meridianus* |  | 1 |
| 4 | *Amara aenea* | 4 | 16 |
| 5 | *Amara chaudoiri* | 1 |  |
| 6 | *Amara convexior* | 2 | 1 |
| 7 | *Amara familiaris* | 1 | 38 |
| 8 | *Amara fulvipes* | 3 |  |
| 9 | *Amara lucida* |  | 2 |
| 10 | *Amara lunicollis* | 27 | 2 |
| 11 | *Amara ovata* | 1 | 3 |
| 12 | *Amara similata* | 1 | 3 |
| 13 | *Amara tricuspidata* | 5 |  |
| 14 | *Anchomenus dorsalis* | 65 | 1046 |
| 15 | *Anisodactylus binotatus* | 1 | 5 |
| 16 | *Anisodactylus nemorivagus* | 2 | 1 |
| 17 | *Anisodactylus signatus* | 1 | 1 |
| 18 | *Asaphidion flavipes* |  | 1 |
| 19 | *Badister bullatus* | 5 | 12 |
| 20 | *Badister sodalis* | 1 | 4 |
| 21 | *Bembidion lampros* | 35 | 70 |
| 22 | *Bembidion obtusum* | 7 | 23 |
| 23 | *Bembidion properans* | 5 | 3 |
| 24 | *Brachinus crepitans* | 6 | 309 |
| 25 | *Brachinus explodens* | 13 | 477 |
| 26 | *Carabus coriaceus* | 2 | 1 |
| 27 | *Carabus germarii* | 3 | 3 |
| 28 | *Carabus granulatus* | 1 | 1 |
| 29 | *Carabus scheidleri* | 1 |  |
| 30 | *Carabus ullrichii* | 4 |  |
| 31 | *Clivina fossor* | 4 |  |
| 32 | *Diachromus germanus* | 21 | 6 |
| 33 | *Drypta dentata* | 1 | 2 |
| 34 | *Harpalus affinis* | 2 | 39 |
| 35 | *Harpalus caspius* | 1 | 3 |
| 36 | *Harpalus distinguendus* |  | 90 |
| 37 | *Harpalus latus* | 8 |  |
| 38 | *Harpalus luteicornis* | 9 | 3 |
| 39 | *Harpalus rubripes* | 11 | 5 |
| 40 | *Harpalus tardus* |  | 1 |
| 41 | *Lebia cyanocephala* |  | 2 |
| 42 | *Microlestes maurus* |  | 2 |
| 43 | *Microlestes minutulus* | 1 |  |
| 44 | *Molops elatus* | 1 |  |
| 45 | *Nebria brevicollis* | 11 | 8 |
| 46 | *Notiophilus aestuans* |  | 3 |
| 47 | *Ophonus azureus* |  | 15 |
| 48 | *Ophonus diffinis* |  | 2 |
| 49 | *Poecilus cupreus* | 55 | 858 |
| 50 | *Poecilus versicolor* | 6 | 10 |
| 51 | *Pseudophonus rufipes* | 9 | 24 |
| 52 | *Pterostichus longicollis* | 2 | 2 |
| 53 | *Pterostichus melanarius* | 14 | 126 |
| 54 | *Pterostichus melas* | 8 | 1 |
| 55 | *Pterostichus ovoideus* | 3 | 5 |
| 56 | *Pterostichus vernalis* | 1 | 7 |
| 57 | *Syntomus obscuroguttatus* |  | 4 |
| 58 | *Trechus quadristriatus* | 1 | 6 |
|  | **Total** | **367** | **3248** |
| **Spiders** | |  |  |
| 1 | *Agyneta mollis* |  | 1 |
| 2 | *Agyneta rurestris* | 1 | 9 |
| 3 | *Alopecosa cf. pulverulenta* | 2 |  |
| 4 | *Alopecosa cuneata* | 15 | 1 |
| 5 | *Alopecosa pulverulenta* | 19 | 40 |
| 6 | *Alopecosa sp.* | 4 | 1 |
| 7 | *Alopecosa trabalis* | 2 |  |
| 8 | *Apostenus fuscus* |  | 1 |
| 9 | *Araeoncus humilis* | 1 |  |
| 10 | *Araneidae gen. sp.* |  | 1 |
| 11 | *Arctosa leopardus* |  | 1 |
| 12 | *Argenna subnigra* | 5 | 3 |
| 13 | *Aulonia albimana* | 3 |  |
| 14 | *Bathyphantes gracilis* |  | 11 |
| 15 | *Bathyphantes parvulus* | 4 |  |
| 16 | *Centromerita bicolor* | 7 | 2 |
| 17 | *Civizelotes gracilis* | 3 | 4 |
| 18 | *Dicymbium nigrum brevisetosum* | 17 | 27 |
| 19 | *Diplostyla concolor* | 3 | 3 |
| 20 | *Drassyllus cf. pusillus* | 5 | 2 |
| 21 | *Drassyllus lutetianus* | 2 | 3 |
| 22 | *Drassyllus praeficus* | 10 | 8 |
| 23 | *Drassyllus pusillus* | 39 | 60 |
| 24 | *Drassyllus sp.* | 6 | 6 |
| 25 | *Enoplognatha thoracica* |  | 1 |
| 26 | *Erigone atra* |  | 4 |
| 27 | *Erigone dentipalpis* | 8 | 27 |
| 28 | *Euophrys frontalis* | 1 |  |
| 29 | *Hahnia nava* | 52 | 9 |
| 30 | *Histopona luxurians* |  | 1 |
| 31 | *Histopona sp.* | 1 |  |
| 32 | *Histopona torpida* | 3 |  |
| 33 | *Inermocoelotes inermis* | 2 |  |
| 34 | *Linyphiidae gen. sp.* | 5 | 79 |
| 35 | *Lycosidae gen. sp.* | 1 | 5 |
| 36 | *Lycosidae gen. sp. (Pulli)* | 66 | 5 |
| 37 | *Mermessus trilobatus* | 25 | 32 |
| 38 | *Micaria pulicaria* | 5 | 8 |
| 39 | *Micrargus subaequalis* | 1 |  |
| 40 | *Oedothorax apicatus* | 2 | 1409 |
| 41 | *Ozyptila simplex* | 3 | 2 |
| 42 | *Ozyptila sp.* | 6 |  |
| 43 | *Pachygnatha cf. degeeri* |  | 1 |
| 44 | *Pachygnatha clercki* |  | 1 |
| 45 | *Pachygnatha degeeri* | 445 | 1484 |
| 46 | *Pachygnatha sp.* | 3 | 1 |
| 47 | *Palliduphantes pallidus* | 1 |  |
| 48 | *Palliduphantes pillichi* | 4 |  |
| 49 | *Pardosa agrestis* | 11 | 344 |
| 50 | *Pardosa alacris* | 4 | 4 |
| 51 | *Pardosa amentata* |  | 1 |
| 52 | *Pardosa cf. agrestis* | 4 | 24 |
| 53 | *Pardosa cf. palustris* | 284 | 113 |
| 54 | *Pardosa cf. prativaga* | 4 | 6 |
| 55 | *Pardosa cf. pullata-Gruppe* | 2 | 1 |
| 56 | *Pardosa hortensis* |  | 3 |
| 57 | *Pardosa lugubris* | 1 |  |
| 58 | *Pardosa paludicola* | 18 | 25 |
| 59 | *Pardosa palustris* | 808 | 439 |
| 60 | *Pardosa prativaga* | 4 | 38 |
| 61 | *Pardosa pullata* | 132 | 66 |
| 62 | *Pardosa sp.* | 9 | 24 |
| 63 | *Phlegra fasciata* |  | 1 |
| 64 | *Phrurolithus festivus* | 2 |  |
| 65 | *Piratula hygrophila* |  | 1 |
| 66 | *Pisaura mirabilis* | 2 | 3 |
| 67 | *Porrhomma errans* | 1 |  |
| 68 | *Porrhomma microphthalmum* |  | 4 |
| 69 | *Porrhomma microps* | 2 |  |
| 70 | *Porrhomma oblitum* |  | 3 |
| 71 | *Robertus arundineti* | 1 | 4 |
| 72 | *Robertus lividus* | 1 | 34 |
| 73 | *Tegenaria campestris* |  | 1 |
| 74 | *Tegenaria s.lat. sp.* | 1 |  |
| 75 | *Tenuiphantes sp. (cf.)* |  | 3 |
| 76 | *Tenuiphantes tenuis* | 2 | 15 |
| 77 | *Thanatus arenarius* |  | 1 |
| 78 | *Thanatus formicinus* | 2 |  |
| 79 | *Tibellus oblongus* |  | 2 |
| 80 | *Tiso vagans* | 4 | 1 |
| 81 | *Trachyzelotes pedestris* | 16 | 12 |
| 82 | *Trochosa ruricola* | 112 | 227 |
| 83 | *Trochosa sp.* | 12 | 16 |
| 84 | *Trochosa terricola* | 28 | 11 |
| 85 | *Walckenaeria vigilax* |  | 12 |
| 86 | *Xerolycosa cf. miniata* | 1 | 1 |
| 87 | *Xerolycosa miniata* |  | 1 |
| 88 | *Xysticus acerbus* | 5 | 11 |
| 89 | *Xysticus bifasciatus* | 3 |  |
| 90 | *Xysticus cf. cristatus-Gruppe* | 1 | 1 |
| 91 | *Xysticus cristatus* | 4 | 5 |
| 92 | *Xysticus kempeleni* |  | 2 |
| 93 | *Xysticus kochi* | 13 | 50 |
| 94 | *Xysticus sp.* | 4 | 1 |
| 95 | *Zelotes latreillei* | 10 | 2 |
| 96 | *Zelotes s.lat. sp.* | 9 | 6 |
| 97 | *Zora spinimana* | 1 |  |
|  | **Total** | **2300** | **4772** |
| **Wild bees** | |  |  |
| 1 | *Andrena bicolor* |  | 1 |
| 2 | *Andrena cineraria* | 1 | 3 |
| 3 | *Andrena curvana* |  | 9 |
| 4 | *Andrena flavipes* | 2 | 19 |
| 5 | *Andrena fulvago* | 3 |  |
| 6 | *Andrena gelriae* |  | 2 |
| 7 | *Andrena gravida* | 4 | 2 |
| 8 | *Andrena haemorrhoa* |  | 1 |
| 9 | *Andrena hattorfiana* | 2 | 9 |
| 10 | *Andrena impunctata* | 3 |  |
| 11 | *Andrena labialis* |  | 1 |
| 12 | *Andrena minutula* |  | 3 |
| 13 | *Andrena minutuloides* |  | 1 |
| 14 | *Andrena nanula* |  | 1 |
| 15 | *Andrena nitidiuscula* |  | 1 |
| 16 | *Andrena ovatula* | 1 | 3 |
| 17 | *Andrena proxima* |  | 1 |
| 18 | *Andrena schencki* |  | 2 |
| 19 | *Andrena strohmella* |  | 5 |
| 20 | *Andrena subopaca* |  | 4 |
| 21 | *Andrena taraxaci* | 2 | 1 |
| 22 | *Anthidium byssinum* | 1 | 2 |
| 23 | *Anthidium oblongatum* |  | 1 |
| 24 | *Anthophora aestivalis* |  | 2 |
| 25 | *Anthophora plumipes* | 1 |  |
| 26 | *Ceratina chalybea* |  | 1 |
| 27 | *Ceratina cyanea* | 1 |  |
| 28 | *Colletes daviesanus* | 1 |  |
| 29 | *Colletes marginatus* |  | 1 |
| 30 | *Eucera longicornis* |  | 2 |
| 31 | *Eucera nigrescens* | 4 | 3 |
| 32 | *Eucera pollinosa* |  | 2 |
| 33 | *Halictus confusus* |  | 3 |
| 34 | *Halictus eurygnathus* | 1 | 2 |
| 35 | *Halictus quadricinctus* |  | 2 |
| 36 | *Halictus scabiosae* |  | 1 |
| 37 | *Halictus sexcinctus* |  | 5 |
| 38 | *Halictus simplex* | 19 | 84 |
| 39 | *Halictus subauratus* | 1 | 5 |
| 40 | *Halictus tumulorum* | 2 | 6 |
| 41 | *Heriades crenulatus* | 2 | 1 |
| 42 | *Heriades truncorum* | 1 | 3 |
| 43 | *Hylaeus communis* | 4 |  |
| 44 | *Hylaeus cornutus* |  | 1 |
| 45 | *Hylaeus sinuatus* |  | 1 |
| 46 | *Lasioglossum angusticeps* |  | 2 |
| 47 | *Lasioglossum calceatum* | 3 | 4 |
| 48 | *Lasioglossum discum* | 1 | 1 |
| 49 | *Lasioglossum fulvicorne* | 1 | 1 |
| 50 | *Lasioglossum glabriusculum* | 4 | 10 |
| 51 | *Lasioglossum lativentre* | 8 | 13 |
| 52 | *Lasioglossum leucozonium* | 3 | 3 |
| 53 | Lasioglossum malachurum | 3 | 5 |
| 54 | *Lasioglossum nigripes* | 1 |  |
| 55 | *Lasioglossum pauxillum* | 5 | 19 |
| 56 | *Lasioglossum puncticolle* | 2 |  |
| 57 | *Lasioglossum villosulum* | 9 | 1 |
| 58 | *Lasioglossum zonulum* |  | 2 |
| 59 | *Megachile ericetorum* |  | 1 |
| 60 | *Megachile ligniseca* |  | 1 |
| 61 | *Megachile willughbiella* |  | 2 |
| 62 | *Melitta tricincta* |  | 2 |
| 63 | *Osmia bicornis* | 1 |  |
| 64 | *Osmia leaiana* |  | 1 |
| 65 | *Osmia leucomelana* | 2 |  |
| 66 | *Sphecodes ephippius* |  | 1 |
|  | **Total** | **99** | **266** |

Table S3. Results from the PERMANOVA (‘adonis’) among studied habitats (newly established grassland and old grassland) based on Bray-Curtis dissimilarity with 999 permutations.

| **Plants** | 2017 |  | Df | Sum Sq | Mean Sq | F | P | **Carabids** | 2017 |  | | Df | | Sum Sq | | Mean Sq | | F | | P | |  |
| --- | --- | --- | --- | --- | --- | --- | --- | --- | --- | --- | --- | --- | --- | --- | --- | --- | --- | --- | --- | --- | --- | --- |
|  |  | Habitat | 1 | 0.943 | 0.943 | 18.25 | **0.012** |  |  | Habitat | | 1 | | 0.429 | | 0.429 | | 2.62 | | **0.008** | |  |
|  |  | Residuals | 8 | 0.413 | 0.051 |  |  |  |  | Residuals | | 8 | | 1.305 | | 0.163 | |  | |  | |  |
|  |  | Total | 9 | 1.357 |  |  |  |  |  | Total | | 9 | | 1.735 | |  | |  | |  | |  |
|  | 2018 |  |  |  |  |  |  |  | 2018 |  | |  | |  | |  | |  | |  | |  |
|  |  | Habitat | 1 | 0.704 | 0.704 | 13.28 | **0.008** |  |  | Habitat | | 1 | | 0.482 | | 0.482 | | 3.50 | | **0.011** | |  |
|  |  | Residuals | 8 | 0.424 | 0.053 |  |  |  |  | Residuals | | 8 | | 1.101 | | 0.137 | |  | |  | |  |
|  |  | Total | 9 | 1.129 |  |  |  |  |  | Total | | 9 | | 1.583 | |  | |  | |  | |  |
|  | 2019 |  |  |  |  |  |  |  | 2019 |  | |  | |  | |  | |  | |  | |  |
|  |  | Habitat | 1 | 0.692 | 0.692 | 11.05 | **0.009** |  |  | Habitat | | 1 | | 0.208 | | 0.208 | | 0.93 | | 0.508 | |  |
|  |  | Residuals | 8 | 0.501 | 0.062 |  |  |  |  | Residuals | | 8 | | 1.783 | | 0.222 | |  | |  | |  |
|  |  | Total | 9 | 1.194 |  |  |  |  |  | Total | | 9 | | 1.992 | |  | |  | |  | |  |
| **Spiders** | 2017 |  |  |  |  |  |  | **Wild bees** | 2017 | |  | |  | |  | |  | |  | |  | |
|  |  | Habitat | 1 | 0.542 | 0.542 | 8.17 | **0.01** |  |  | | Habitat | | 1 | | 0.786 | | 0.786 | | 4.00 | | **0.017** | |
|  |  | Residuals | 8 | 0.531 | 0.066 |  |  |  |  | | Residuals | | 7 | | 1.374 | | 0.196 | |  | |  | |
|  |  | Total | 9 | 1.074 |  |  |  |  |  | | Total | | 8 | | 2.161 | |  | |  | |  | |
|  | 2018 |  |  |  |  |  |  |  | 2018 | |  | |  | |  | |  | |  | |  | |
|  |  | Habitat | 1 | 0.410 | 0.410 | 4.70 | **0.01** |  |  | | Habitat | | 1 | | 0.631 | | 0.631 | | 2.09 | | **0.005** | |
|  |  | Residuals | 8 | 0.698 | 0.087 |  |  |  |  | | Residuals | | 8 | | 2.406 | | 0.300 | |  | |  | |
|  |  | Total | 9 | 1.109 |  |  |  |  |  | | Total | | 9 | | 3.038 | |  | |  | |  | |
|  | 2019 |  |  |  |  |  |  |  | 2019 | |  | |  | |  | |  | |  | |  | |
|  |  | Habitat | 1 | 0.313 | 0.3136 | 4.66 | **0.008** |  |  | | Habitat | | 1 | | 0.600 | | 0.600 | | 2.02 | | **0.014** | |
|  |  | Residuals | 8 | 0.538 | 0.0672 |  |  |  |  | | Residuals | | 8 | | 2.367 | | 0.295 | |  | |  | |
|  |  | Total | 9 | 0.851 |  |  |  |  |  | | Total | | 9 | | 2.967 | |  | |  | |  | |


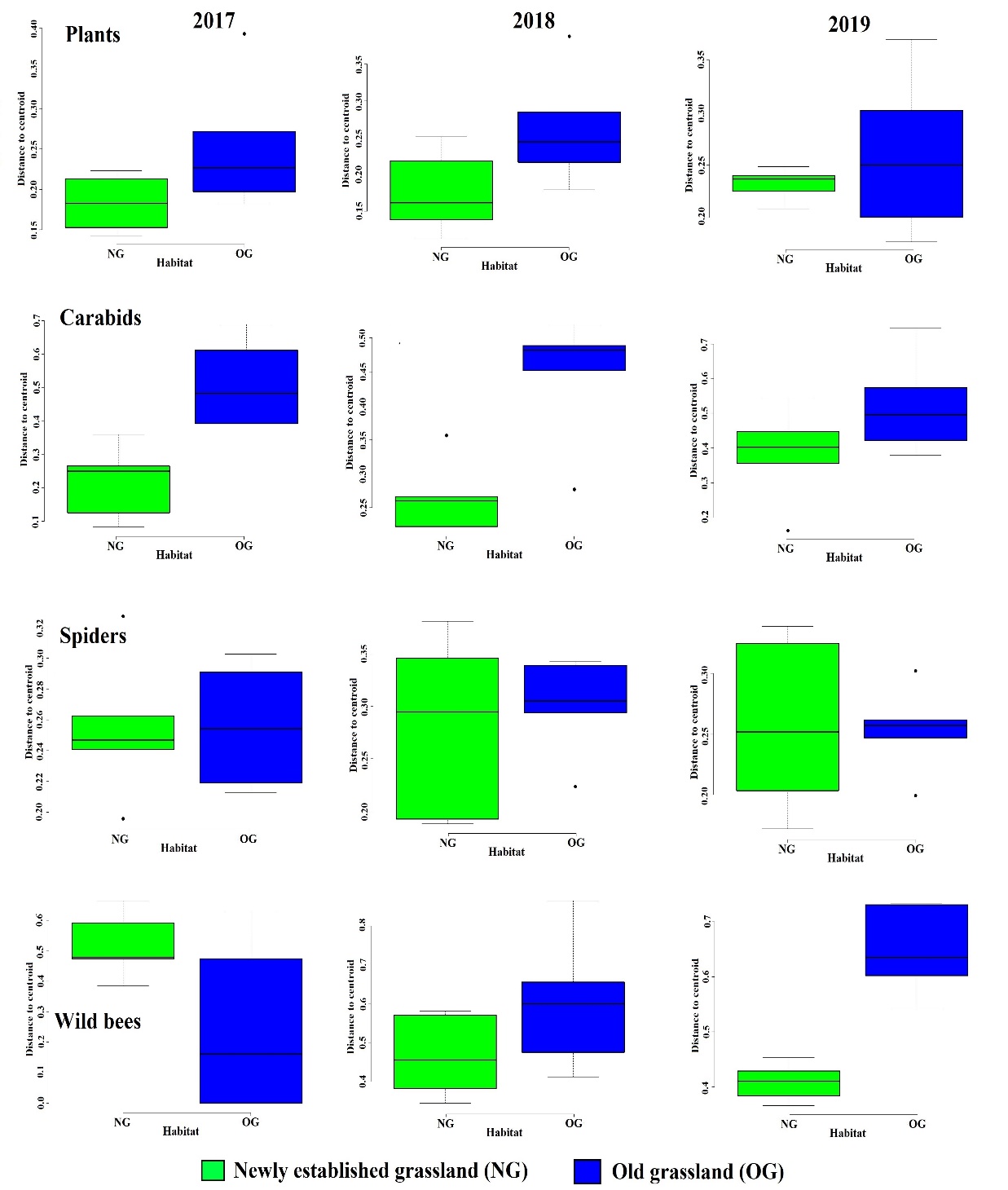


Figure S1. Box and whiskers plot showing the variation in the distribution of distance to centroid of pairwise comparisons among newly established grassland (NG) and old grassland (OG).
